# Prevalence of Depression among Iranian Elderly: Systematic Review and Meta-Analysis

**Published:** 2018-01

**Authors:** Diana Sarokhani, Masoumeh Parvareh, Ali Hasanpour Dehkordi, Kourosh Sayehmiri, Abbas Moghimbeigi

**Affiliations:** 1 Psychosocial Injuries Research Center, Ilam University of Medical Sciences, Ilam, Iran.; 2 Social Determinants of Health Research Center, Shahrekord University of Medical Sciences, Shahrekord, Iran.; 3 Department of Medical-Surgical, Faculty of Nursing and Midwifery, Shahrekord University of Medical Sciences, Shahrekord, Iran.; 4 Department of Biostatistics, Psychosocial Injuries Research Center, Ilam University of Medical Sciences, Ilam, Iran.; 5 Modeling of Noncommunicable Diseases Research Center, Department of Biostatistics, School of Public Health, Hamadan University of Medical Sciences, Hamadan, Iran.

**Keywords:** *Depression*, *Elderly*, *Iran*, *Prevalence*

## Abstract

**Objective:** depression is one of the most serious and prevalent mood disorders. Aging population is an important economic, social, and health challenge of the 21^st^ century. The present study aimed at estimating the prevalence of depression among the Iranian elderly through meta-analysis method.

**Method** **:** Studies were searched in ISI, Scopus, Pub Med, Google Scholar, and in Iranian databases including Iran Medex, Magiran, SID, and Med Lib using the following keywords: “depression”, “prevalence”, and “elderly”. Data were analyzed using meta-analysis (random effects model). Heterogeneity among the results of the studies was examined by “I^2^” index. Beck, DASS-21, GHQ-28, and G DS questionnaires were used in this study, and analyses were performed using STATA Ver.11.

**Results:** A total of 26 studies in Iran with a sample size of 5781 individuals had been found during 2001 and 2015. Prevalence of depression among Iranian elderly was estimated to be 43% (95% confidence interval (CI):30% - 55%). The findings showed that the prevalence of depression among Iranians were49% in women, 48% in men, 37% in unmarried, and 45%in the married. In addition, the prevalence of very severe, severe, moderate, and mild depression levels were estimated to be 5%, 19%, 33%, and 38% of the participants, respectively. No significant difference was observed between married and unmarried individuals. Most of Iranian elderly suffered from mild depression.

**Conclusion: **There was high level of depression prevalence among Iranian elderly, and women were more depressed than men. So, policy makers must design and run mental health programs to decrease the prevalence of depression among Iranian elderly.

Depression has been one of the most common disorders in Iran, the world's fourth costly impairment, and undoubtedly the second costliest disorder until 2015. Some of the demographic and social characteristics among the elderly reduce or increase their depression (1). Depression is a public issue and constitutes 35% to 45%of mental illnesses in Iran (2) and generally covers about 8% to 20% of the population (3). 

The chronic nature of some diseases has many consequences on patients' lives especially the quality of lives, duration of treatment, mortality, new admissions, and the cost of hospital readmissions (4-9).

Aging is a stage of life that is naturally associated with reduced physical and mental abilities (10-12). 

So, it will be one of the most important health problems of societies in the future. Psychological problems, particularly depression, are the most common problems in this age group (13). 

In addition, aging is a term used for lack of ability to effectively reduce biological life along with aging (14). Along with aging, problems and depression in the elderly have increased, making aging the most common mental disorder (15). 

Mental health is one of the key areas of elderly individuals’ health aspects and requires particular attention to prevent such disorders as depression and anxiety. For several reasons, elderly people are much vulnerable considering mental health, and about 15% to 25%of the elderly have serious mental health problems, so that depressive disorders increase with each decade of age increase (16). Depression and cognitive impairment are common problems in the elderly that reduce performance, quality of life, and increase mortality (17). Depressive disorders are one of the most common psychiatric disorders of the 20th century, particularly in nursing homes with a prevalence of 43% to 86%. The disease increases the risk of suicide and health care costs (18). Depression can be prevented and treated in the elderly, and eliminating it improves quality of life (19). 

The most common symptoms of depression in the elderly occur in the form of lethargy, fatigue, lack of concentration, irritability, lack of hearts and minds, frequent waking from sleep, loss of appetite, and physical pain (20). Loneliness has been noted as a sign of depression and an independent risk factor in psychological damages of aging, and the elderly experience it for various reasons such as physical defects, loss of relatives, and reduced communication (21). Social isolation and loneliness are among the factors affecting the incidence of depression and dementia in the elderly (22). Also, moderate and severe hearing loss in the elderly can affect their physical and mental-social health. These people may suffer from poor self-esteem, irritability, isolation, frustration, depression, and probably amnesia (23). The rate of depression in elderly men has been mostly affected by their physical health and mobility problems. Also, elderly women show more symptoms of depression and anxiety due to receiving less social and family support (24).

Considering numerous studies done on the prevalence of depression among the elderly in Iran, conducting a meta-analysis study is necessary to validate the results of these studies, so that a precise and valid rate is provided for planners and researchers in the field. Thus, this study aimed at estimating the prevalence of depression in the elderly in Iran using systematic review and meta-analysis. This study conducted a systematic review of previous studies, performed meta-analysis of the final data, and finally assessed the prevalence of depression in the elderly in Iran.

## Materials and Methods

*Search Strategy*

The reviewed articles were collected through searches in the internet and manual search. Databases including Iran Medex, SID, Magiran, Irandoc, Medlib, IranPsych, Science Direct, ISI, PubMed, and Scopus were searched. All articles, theses, national and international scientific journals, papers presented at congresses and organizational reports were searched up to December 2015. 

To gain high sensitivity, search was done in Iranian databases only through keywords of “Prevalence”, “Depression”, “Elders”, and “Iran” because some websites did not show sensitivity to the search operators (OR, AND, NOT). However, international databases were searched through the keywords of "Iran", "Elders", "Prevalence", and" Depression". The keywords were standard in MeSH and eventually “Depression AND Elders” strategy was used to search. In addition, reference lists of the selected articles were evaluated for finding relevant studies. 

*Study Selection*

First, a list of titles and abstracts of all searched papers in national databases was prepared by 2 researchers independently. Then, duplicated article titles were excluded. Next, articles’ abstracts were reviewed to find appropriate studies. Study selection in international databases was similar to that of national databases.

Study inclusion criteria were as follow: (1) elderly population, (2) referring to prevalence of depression in the elderly in Iran, (3) and studies conducted in the last 14 years. Minimum entry criteria were used to increase the sensitivity of article selection. However, to find the most relevant and highest quality studies, exclusion criteria were as follows: (1) non-related studies in study method and research topic, and (2) studies which did not have enough information. The low quality of studies was assessed through the STROBE checklist (Strengthening the reporting of observational studies in epidemiology) (25). 

*Data Extraction*

To reduce bias in reporting and error in data collection, 2 researchers independently extracted data using a standardized data collection form that had already been prepared. The form was first designed by the study team and included the following items: author’s name, title of study, year of publication, journal name, study design, sample size, city, questionnaire type, and prevalence of depression.

This study included 3 questionnaires. The first was the Geriatric Depression Scale (GDS) Questionnaire, which was designed and certified to evaluate depression in the elderly (26). This questionnaire is simple and easy and is used to rank the elderly in three levels of severe, moderate, and mild depression (27). All the questions are yes-no. The questionnaire is an appropriate scale to assess symptoms of depression in the elderly and has been validated in multiple clinical and non-clinical environments (21). 

Beck's Depression Inventory (BDI) Questionnaire was the second questionnaire in the study to assess depression in the elderly (23). It has 21 multiple-choice questions, which is graded from 0 to 3 scores and determines the severity of an individual’s depression (1). The questionnaire covers various aspects of depression and its purpose is to measure depression (28). 

The third questionnaire was Kessler test which measures anxiety and depressive disorders. The questionnaire assesses signs and symptoms of anxiety and depression in the 4 weeks prior to questioning, and it covers the feelings of sadness, nervousness, restlessness, hopelessness, effort, and worthlessness (29). 

Goldberg Health Questionnaire (GHQ) was the last questionnaire in the study, which is used in many studies, and its validity and reliability has been confirmed (20). The 28-item version of Goldberg’s Questionnaire has domains for psychopathology subscales. In addition to total score, it has scores for the 4 scales of somatization of symptoms, anxiety, social dysfunction disorder, and depression, each having 7 questions (30).

*Statistical Analysis*

Distribution of prevalence of depression was binomial, so its variance was computed using binomial distribution. The effect size (ES) in this study was defined as prevalence of depression. Due to significant heterogeneity between studies (P=0.000), random-effects model in meta-analysis was used to combine the results of studies. Meta-regression was used to find a relationship among prevalence of depression among the elderly and sample size and year of study. Sensitivity analysis was used to evaluate the effect of each study on pooled analysis. Funnel plot and Beggs test were used to assess publication bias. Data were analyzed using STATA Ver. 11 software.

## Results

*Inclusion Method Summary of Studies to the Meta-analysis*

**Chart 1:**
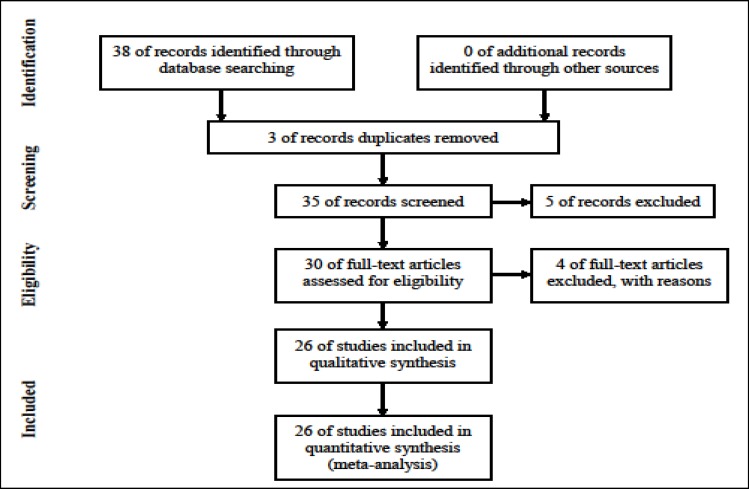
Flowchart of Steps Involved in Entering the Studies into the Systematic Review and Meta Analysis Process

In the first phase of the search, 38 articles were selected and after reviewing the titles, 3 duplicates and overlapping articles were excluded. Abstracts of 35 possibly relevant articles were reviewed and 5 other irrelevant articles were identified and excluded. The full-texts of the remaining 30 articles were reviewed. Moreover, 4studies were deleted because of lack of sample size for depression, and finally, 26 articles were accepted for inclusion in the meta-analysis (Chart 1).

**Figure1:**
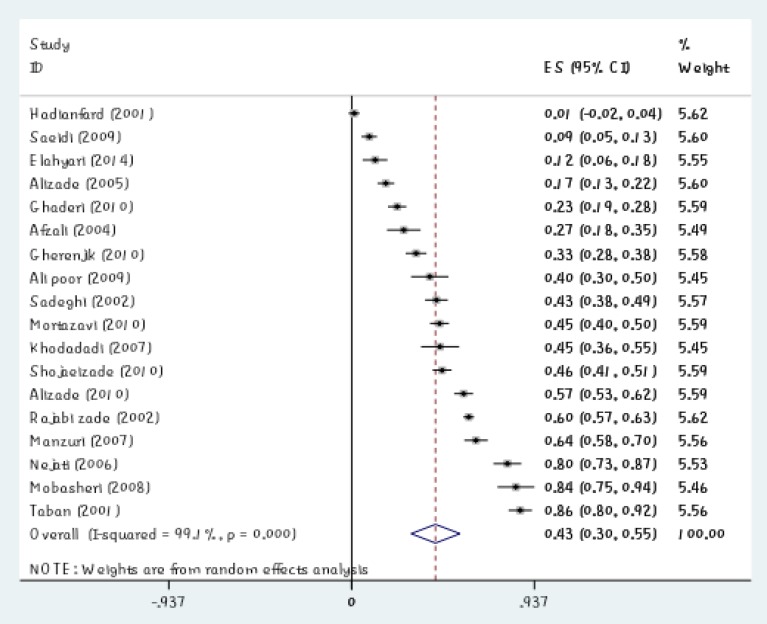
Prevalence of Depression in Iranian Elderly and its 95% CI in Iran Based on Author’s Name and Year of Study According to Random Effects Model

In 26 studies with a sample of 5781 individuals, 18 articles reported the prevalence of depression among the elderly in Iran. Prevalence of depression among the elderly in Iran was estimated to be 43% (95% CI, 30%-56%). In this study, the lowest prevalence of depression in Iranian elderly was 1% in the study of Hadianfard et al. in 2001 (95% CI, - 2%-4%) and the highest prevalence of depression among Iranian elderly was 86% in the study of Taban et al. in 2001 (95% CI, 80%-92%) (Table 1). Due to the heterogeneity among studies, random-effects model was used to compute pooled estimate of prevalence of depression in the elderly (Figure 1).

Prevalence of depression was estimated to be 49% (95% CI, 18%-81%) in elderly women, 48% (95% CI, 16%-79%) in elderly men, 37%(95% CI, 15%-60%) in single elderly people, and 45%(95% CI, -1%-92%) in married elderly. Also, the prevalence of very severe depression was 5% (95% CI, 2%-8%) in the elderly, severe depression 19% (95% CI, 12%-25%), moderate depression 33% (95% CI, 25%-41%), and mild depression 38% (95% CI, 29%-46%) (Table 2).

In an analysis done on 9 studies conducted on the elderly population in the northern Iran, prevalence of depression in the elderly was estimated to be 42% (CI 95%, 30%-55%),and it was found to be 35%(95% CI, 15%-84%) in 2 studies conducted in southern Iran. The prevalence was 51 %( 95% CI, 19%-83%) in 6 studies in central Iran. There was only 1 study in other regions of Iran. In the analysis that was done by the type of questionnaire, the prevalence of depression in Iranian elderly, using Beck questionnaire, was 49%(95% CI, 21%-77%) in 4 studies, 48%(95% CI, 28%-68%) for GDS Questionnaire in 8 studies, 40%(95% CI, 16%-64%) for Kessler questionnaire in 3 studies, and there was only 1 study from other questionnaires. 

**Figure 2:**
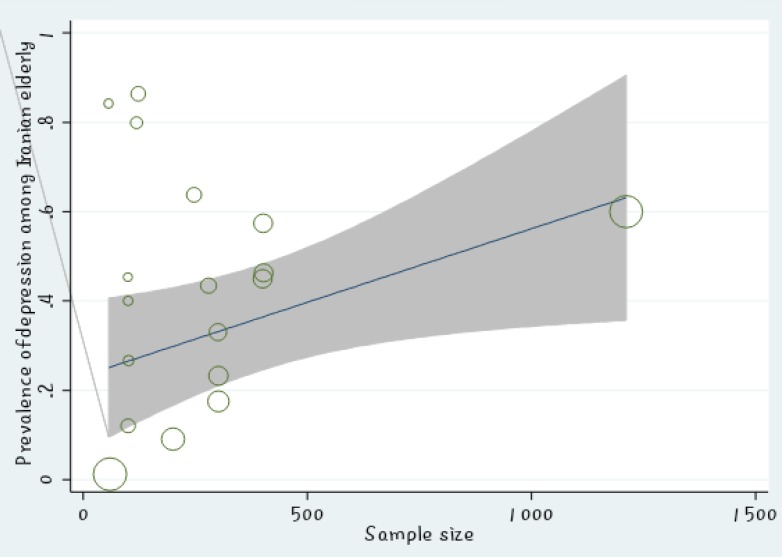
The Relationship between Prevalence of Depression in Iranian Elderly and Sample Size Using Meta-regression

**Figure 3:**
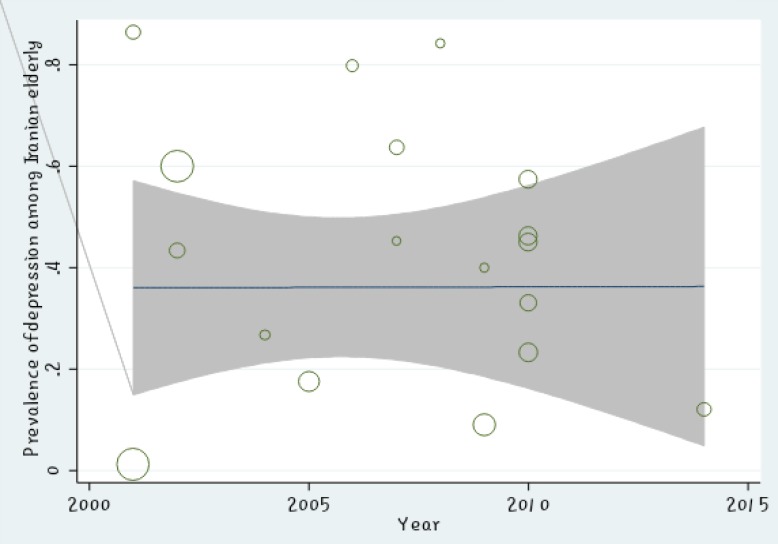
The Relationship between Prevalence of Depression in Iranian Elderly and Year of Study Using Meta-regression

**Figure 4:**
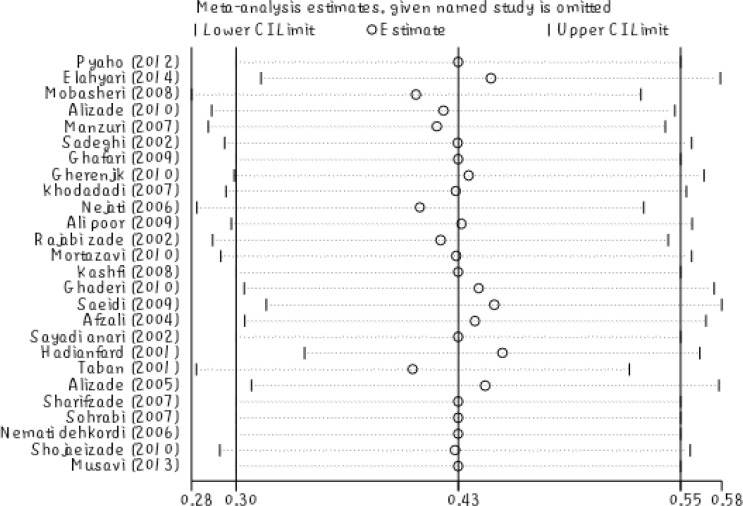
Sensitivity Analysis

**Figure5:**
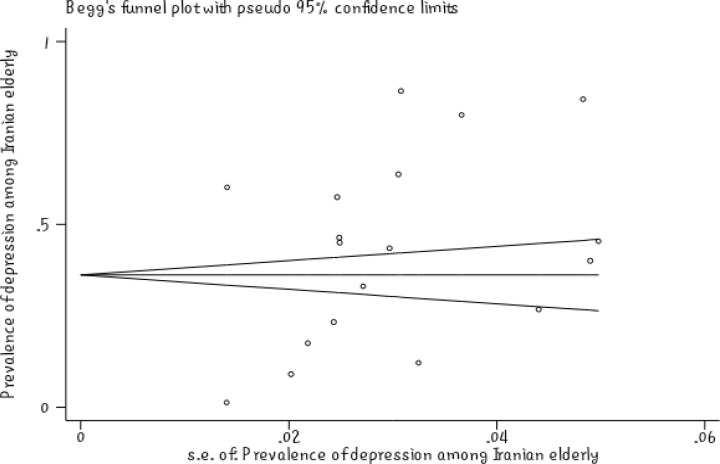
Publication Bias

The meta-regression showed no significant relationship between the prevalence of depression in Iranian elderly and the sample size (P =0.447) (Figure 2). Also, there was no significant relationship between the prevalence of depression in Iranian elderly and the year of study (P =0.596) (Figure 3). Sensitivity analysis was used to assess the effect of each study on the final result (Figure 4). Funnel figure of Beggs test showed that the effect of publication bias was not significant (P=0.430) (Figure 5).

The midpoint of each segment showed the prevalence of depression in elderly in each study. Rhombus shape indicated the prevalence of depression in elderly in Iran in all studies.

## Discussion

A total of 26 articles were reviewed with a sample size of 5781 individuals, and 18 articles reported the prevalence of depression in Iranian elderlies. The prevalence of depression among the elderly in Iran was estimated to be 43% (95% CI, 30%-56%). In this study, the lowest prevalence of depression in Iranian elderly was 1% in the study of Hadianfard and the highest prevalence of depression among Iranian elderly was 86% in the study of Taban et al. 

The prevalence of depression was 49% in elderly women, 48% in elderly men, 37% in the single elderly, and 45% in the married elderly. These results indicated that depression has a higher prevalence among married individuals than men and the singles. Mortazavi performed a study in 2010 and estimated the prevalence of depression to be 52.8% in elderly women and 31.8% in elderly men (30). Multiple assessments have reported higher depression prevalence in women than in men (31), and these results are consistent with our study.

Also, the prevalence of very severe depression was 5% in the elderly, severe depression 19%, moderate depression 33%, and mild depression 38%. Hence, most of the Iranian elderly suffer from mild depression. A study reported 64.6% prevalence for depression in the elderly; moreover, 51.5% of the participants had moderate depression and 13.1% had severe depression (32). In another study, the prevalence of mild depression in the elderly was 16.8% and major depression was 14.7% (33). Qaranjik studied elderly Turkmens in 2010 and reported the prevalence of moderate depression to be 10% and severe depression to be 3% (34), and these results are consistent with our study.

In the analysis done by the type of questionnaire, the prevalence of depression in Iranian elderly using Beck questionnaire was 49%; it was 48% for GDS questionnaire, and 40% for Kessler questionnaire. So, studies that used Beck questionnaires had the highest of depression prevalence. In the analysis done based on Iran’s regions, the prevalence of depression in the elderly people in northern Iran was 42%, it was 35% in southern Iran, and 51% in central Iran. The results of these studies revealed that the elderly in central Iran had the highest prevalence of depression. 

Based on the meta-regression figure, there was no significant relationship between the prevalence of depression in Iranian elderly and the sample size which meant that prevalence of depression in Iranian elderly did not increase by increase in sample size. In Figure 2, the size of the circle shows the largeness of sample size (P =0.447). The meta-regression revealed no significant relationship between the prevalence of depression in Iranian elderly and year of study (P =0.596); moreover, prevalence of depression in Iranian elderly did not increase during 2001 to 2015, the years reviewed in this study (Figure 3).

Circles show relative risk (RR) by removing studies, and segments show 95% CI for RR. This figure shows the effect of the removal of any study on the final outcome of this study. Based on the above figure, prevalence of depression in Iranian elderly increases to 47% by removing the study of Taban in 2011 (95% CI, 35%-50%) and prevalence of depression in Iranian elderly decreases to 28% by removing the study of Mobasheri in 2008 (95% CI, 28%-52%). These two were the most weighted studies in the final result of this research. Funnel plot of Beggs test showed that the effect of publication bias was not significant (P=0.430). It is better if the figure is not significant because it shows that the studies that have been done in this area had a chance of being published and have been usually published (Figure 5). 

Epidemiological studies conducted in this field in Iran are limited. Taban (2002) reported the prevalence of depression in the elderly in nursing homes in Isfahan to be 86% (35). In another study, the prevalence of 22.4% has been reported for depression and loneliness in the elderly across the country (36). According to the study of Baaseri et al. on 110 nursing home residents in Mashhad, 23.9% of the elderly had a depression score of 5 or more (37). Alahyari et al. performed a study and reported the depression prevalence of 12% for the elderly (38). The prevalence of depression in the elderly in Tehran was 79.8% in the study of Nejati (39). Due to obtaining different results from previous studies, performing a meta-analysis study is necessary. 

The results of a study in Greece showed the prevalence of 27% for mild to moderate depression and 12% for moderate to severe depression (40). In another study in China's rural elderly, 26.5 of them had mild depression and 4.3% had severe depression (41). William (1980) reported the depression prevalence of 2% to 4% in American people over 65 years and the prevalence of 5% to 44% for milder depression (42). In another study on Chinese elderly living in Hong Kong, the prevalence of depression was 11% in men and 14% in women (43).

## Limitation

Due to the different types of questionnaires used in the reviewed articles, the difference in scoring the questions of the respective questionnaires and the difference in the number of questions in questionnaires, we could not combine the results of different questionnaires and report accurate statistics on the prevalence of depression among elderly in general and for various dimensions. Because of the variety of questionnaires, we did not manage to estimate the average score of the prevalence of depression among elderly in terms of age and place of research.

## Conclusion

Given the high prevalence of depression in the elderly and, in particular, the high prevalence of depression in elderly women, the use of interventions including education about symptoms of depression for early diagnosis and timely reference elderly patients are highly essential. Also, due to the high prevalence of mild depression, critical planning is necessary for timely diagnosis and treatment.

Study limitation: The present study had some limitations including lack of access to full-text articles, different measurement tools, and lack of uniform distribution of studies between different regions of the country.

Different measurement tools to estimate prevalence of depression were another limitation of the study. For example, BDI is not a diagnostic test, but a test to evaluate the severity of depression. So, combination of prevalence of these tools must be considered in interpretation of the results.

There was no information about the prevalence of depression in villager elderly, retired, widows, and jobless elderly. Thus, we suggest conducting a large number of original research in these fields to estimate the prevalence of depression in this group.

**Table 1:** Specifications of Articles under Review for Prevalence of Depression among Iranian Elderly

**ID**	**Author**	**Year**	**City**	**Questionnaire**	**Age Average**	**Sample Size**	**Prevalence of Depression in ** **Elderly People (%)**
(27)	Payahoo	2012	Tabriz	GDS	69.4±7.9	184	
(38)	Alahyari	2014	Tehran	Beck		100	12
(31)	Mobasheri	2008	Shahr kord	Beck	68.7±16.1	57	84.2
(24)	Alizadeh	2010	Tehran	Kessler	71.5±8.9	402	57.4
(26)	Manzouri	2007	Esfahan	GDS	60-74	248	63.7
(44)	Sadeghi	2002	Tehran	GDS	65	279	34.4
(45)	Ghafari	2009	Tehran	DASS-21	64.22±4.57	104	
(34)	Gharanjik	2010	Torkman	GDS	68±7.04	300	33
(14)	Khodadady	2007	Rasht	GDS	64.65	100	45.3
(39)	Nejati	2006	Tehran	GDS	60-76	120	79.8
(16)	Alipour	2009	Tehran	Beck	60-70	100	40
(3)	Rajabizadeh	2002	Kerman	Beck	55-64	1212	60
(30)	Mortazavi	2010	Shahrkord	GHQ-28	77	400	45
(1)	Kashfi	2008	Shiraz	Beck	70	120	
(13)	Ghaderi	2010	Bookan	GDS	70. 69	302	23.3
(2)	Saeedi	2009	Ahvaz	GDS	71±8	200	9
(46)	Afzali	2004	Shahrkord		74-65	101	7.26
(47)	Sayadi anari	2002	Toos	Beck	60-75	30	
(48)	Hadianfard	2001	Shiraz	Scl-90-r		60	1.2
(35)	Taban	2001	Esfahan	GDS		124	86.4
(24)	Alizadeh	2009		Kessler	65-69	302	1.75
(49)	Sharifzadeh	2007	Birjand		71 ±7.8	250	
(20)	Sohrabi	2007	Shahrood	GDS	72.39 ±9.11	136	
(50)	Nemati Dehkordi	2006	Shahrkord	GDS	70-75	64	
(51)	Shojaeizadeh	2010	Tehran	Kessler	71.5 ±8.9	402	3.46
(52)	Musavi	2013	Orumie	GDS		84	

**Table 2:** The Prevalence of Depression in the Elderly in the Groups Studied in Iran

**Subgroups**	**Number ** **of study**	**Sample ** **size**	**Min The prevalence ** **of depression in ** **elderly people ** **(95%CI )**	**Max The prevalence ** **of depression in ** **elderly people ** **(95%CI )**	**The prevalence of ** **depression in ** **elderly people ** **(95%CI )**
**The prevalence of ** **depression in elderly ** **people**	18	4809	(55%-30%)43%	(92%-80%)86%	(4%-0%)1%
**The prevalence of ** **depression in elderly ** **women**	6	671	(81%-18%)49%	(90%-76%)83%	(4%-0%)1%
**The prevalence of ** **depression in elderly ** **men**	6	471	(79%-16%)48%	(96%-80%)88%	(8%-0%)4%
**The prevalence of very ** **severe depression in the ** **elderly**	2	11	(8%-2%)5%	(11%-1%)6%	(9%-1%)5%
**The prevalence of severe ** **depression in the elderly**	19	1648	(25%-12%)19%	(97%-87%)92%	(1%-0%)0%
**The prevalence of ** **moderate depression in ** **the elderly**	14	1524	(41%-25%)33%	(81%-46%)63%	(16%-3%)9%
**The prevalence of mild ** **depression in the elderly**	16	1956	(46%-29%)38%	(74%-63%)68%	(13%-3%)8%
**The prevalence of ** **depression in married ** **elderly**	3	1295	(92%-0%)45%	(86%-82%)84%	(20%-5%)13%
**The prevalence of ** **depression in single ** **elderly**	3	275	(60%-15%)37%	(56%-44%)51%	(25%-9-%)8%
